# Serum Klotho as a potential biomarker for prognosis in acute ischemic stroke

**DOI:** 10.3389/fmed.2026.1850285

**Published:** 2026-06-03

**Authors:** Fangfang Bi, Juning Wang, Mingquan Su

**Affiliations:** Xi’an Peihua University, Xi’an, Shaanxi, China

**Keywords:** acute ischemic stroke, biomarker, Klotho, logistic regression, prognosis

## Abstract

**Objective:**

To investigate the association between serum Klotho levels and 90-day functional outcome in patients with acute ischemic stroke (AIS), and to assess its potential as a short-term prognostic biomarker.

**Methods:**

This single-center retrospective observational cohort study included 200 patients with AIS. Patients were divided into favorable outcome group [90-day modified Rankin Scale (mRS) 0–2, *n* = 140] and unfavorable outcome group (mRS 3–6, *n* = 60). Unfavorable 90-day outcome was used as the binary dependent variable. Baseline characteristics were compared between groups. Univariate and multivariable logistic regression analyses were performed to assess the association between serum Klotho and unfavorable outcome. Klotho levels were also analyzed by quartiles with trend testing. Restricted cubic spline analysis was used to evaluate the dose–response pattern. Receiver operating characteristic curves and the DeLong test were used to compare the predictive performance of the Klotho-alone, clinical, and combined models. Sensitivity analyses were conducted to assess robustness.

**Results:**

Serum Klotho levels were significantly lower in patients with unfavorable outcomes than in those with favorable outcomes [579.65 (444.50, 664.12) pg/mL vs. 754.05 (656.00, 817.95) pg/mL, *p* < 0.001]. After adjustment for age, sex, baseline NIHSS score, atrial fibrillation, diabetes, eGFR, hsCRP, time from symptom onset to blood collection, and reperfusion therapy, each 1-standard deviation increase in serum Klotho was associated with a lower risk of unfavorable outcome (*OR* = 0.294, 95% *CI*: 0.158–0.505, *p* < 0.001). Compared with the lowest quartile, higher Klotho quartiles were associated with reduced risk, with a significant trend across quartiles (*p* < 0.001). Restricted cubic spline analysis showed a significant overall association but no significant nonlinear relationship. The AUCs of the Klotho-alone, clinical, and combined models were 0.836, 0.868, and 0.902, respectively, although the improvement over the clinical model was not statistically significant. Sensitivity analyses showed consistent results.

**Conclusion:**

Lower early serum Klotho levels were independently associated with unfavorable 90-day outcome in patients with AIS. However, its incremental predictive value beyond established clinical variables was not statistically confirmed. Further prospective studies are needed to validate its prognostic utility.

## 1 Introduction

Strokes represent one of the principal causes of mortality and morbidity on a global level (1, 2), the majority of which being ischemic strokes (3–5). Although various achievements have been made in the fields of acute reperfusion treatment, stroke units care, and rehabilitation procedures, individual variability persists in the functional status of AIS patients, with a number of patients developing severe disabilities and even dying within 90 days after the occurrence of AIS (6–8). In this regard, the discovery of biomarkers, characterizing the condition of AIS, and indicating risk stratification would be critically important.

Klotho is a transmembrane protein and soluble protein with anti-aging properties (9, 10), primarily expressed in the kidneys, parathyroid glands, and choroid plexus of the brain (11). Previous studies suggest that Klotho may be related to biological aging, vascular function, inflammation, and oxidative stress regulation. However, its role in human cerebrovascular disease remains incompletely defined, and current evidence is mainly derived from population-based association studies, experimental models, and non-stroke-specific research (12, 13). Both basic and translational research suggest that Klotho may affect brain ischemia-induced injury and repair processes by alleviating inflammation, inhibiting oxidative stress, and maintaining the integrity of the blood–brain barrier and neurovascular unit.

Recently, increasing attention has been paid to the relationship between circulating Klotho and cerebrovascular disease. A population-based NHANES study reported that lower serum Klotho levels were independently associated with a higher prevalence of stroke, although the cross-sectional design precluded causal inference (14). In addition, a recent retrospective study of young to middle-aged adults with AIS found that Klotho levels were lower in AIS patients than in stroke-free controls and were inversely associated with AIS risk, suggesting its potential relevance to early risk stratification (12). However, evidence directly evaluating serum Klotho as a prognostic biomarker for functional outcome after AIS remains limited. The potential prognostic relevance and incremental value of Klotho therefore require further investigation. Based on this background, the present study aimed to evaluate the association between serum Klotho levels and 90-day outcomes in patients with AIS.

## 2 Materials and methods

### 2.1 Study design and participants

This was a single-center retrospective observational cohort study. A total of 200 patients with acute ischemic stroke (AIS) who were consecutively admitted to Xi’an Peihua University Hospital between January 2021 and January 2025 were included. Clinical data were retrospectively extracted from the electronic medical record system and follow-up records. The diagnosis of AIS was established according to the Chinese Guidelines for Diagnosis and Treatment of Acute Ischemic Stroke 2018 and updated 2023 versions, based on acute clinical manifestations and cranial CT/MRI findings (15, 16).

Inclusion criteria: (1) age ≥18 years; (2) Meets the diagnostic criteria for AIS; (3) completion of serum Klotho and related clinical data collection after admission; (4) completion of 90-day follow-up and obtaining 90-day outcome data.

Exclusion criteria: (1) coexisting severe infections, malignancies, autoimmune diseases, or other conditions that may significantly affect Klotho levels; (2) missing critical clinical data; (3) loss to follow-up or inability to obtain 90-day outcome data.

These exclusion criteria were applied to reduce potential confounding from systemic diseases that may substantially influence circulating Klotho levels. However, we acknowledge that this approach may limit the representativeness of the study population. Patients with missing serum Klotho measurement, missing 90-day mRS outcome, or missing critical clinical covariates required for the main multivariable analyses were excluded before construction of the final analytic cohort.

This study was approved by the Life Sciences and Medical Research Ethics Committee of Xi’an Peihua University (Approval No. XPHU2025007).

### 2.2 Clinical data collection

Demographic data, medical history, clinical features, laboratory indices, imaging data, and treatment information were collected for all patients. Demographic and general information included age, sex, body mass index (BMI), smoking history, etc. Medical history included hypertension, diabetes, atrial fibrillation, prior stroke, and chronic kidney disease (CKD).

These measures include admission systolic blood pressure, diastolic blood pressure, baseline NIHSS score, ASPECTS score, time from symptom onset to admission, time from symptom onset to blood sample collection, pre-stroke mRS score, and infarct volume. The lab variables are fasting blood glucose (FBG), serum creatinine, estimated glomerular filtration rate (eGFR), high-sensitivity C-reactive protein (hsCRP), and low-density lipoprotein cholesterol (LDL-C). In addition, the TOAST classification, use of intravenous thrombolytic treatment, endovascular intervention, and reperfusion treatment are also recorded, along with clinical events such as early neurological deterioration (END) and symptomatic intracerebral hemorrhage (sICH).

### 2.3 Blood sample collection and Klotho measurement

The sampling window was defined according to hospital admission. For each patient, the serum sample used for Klotho measurement was collected once within 24 h after hospital admission. Venous blood was drawn by trained nurses using standard peripheral venipuncture, and the exact clock time of blood collection was recorded for each patient. To account for variability in the timing of sampling after stroke onset, onset-to-blood collection time was calculated as the interval between symptom onset, or the last-known-well time when the exact onset time was unavailable, and the actual blood draw. Therefore, “within 24 h after admission” referred to the allowable sampling window, whereas onset-to-blood collection time was used as a timing-related covariate in the statistical models. For clinically stable patients admitted before routine morning laboratory testing, fasting morning blood samples were obtained after overnight fasting. For patients requiring urgent diagnostic evaluation or reperfusion treatment, non-fasting samples were collected at the first clinically feasible time within 24 h after admission. Blood samples were allowed to clot at room temperature and were then centrifuged at 3000 rpm for 10 min at 4 °C. The separated serum was aliquoted and stored at −80 °C until batch analysis, and repeated freeze–thaw cycles were avoided.

Serum Klotho concentrations were measured using a commercially available human soluble *α*-Klotho enzyme-linked immunosorbent assay kit (IBL, Gunma, Japan; catalog No. 27998) according to the manufacturer’s instructions. The assay detection range was 93.75–6,000 pg/mL, and the minimum detectable concentration was 6.15 pg/mL. The intra-assay and inter-assay coefficients of variation were <4 and <12%, respectively. All serum samples, standards, and quality-control samples were measured in duplicate, and the mean value of the duplicate measurements was used for statistical analysis. Samples with a duplicate coefficient of variation greater than 15% were remeasured. Serum Klotho was measured once using the first available serum sample collected within 24 h after admission. Serial blood samples were not available; therefore, intra-individual variability and temporal changes in serum Klotho levels during the acute and recovery phases could not be assessed in this study.

### 2.4 Outcome definition

Functional outcome at 90 days was the primary outcome and was assessed using the modified Rankin Scale (mRS). Patients were classified into two groups according to their 90-day mRS scores: favorable 90-day outcome, defined as mRS 0–2, indicating functional independence; and unfavorable 90-day outcome, defined as mRS 3–6, indicating functional dependency or death. In the binary logistic regression analyses, unfavorable 90-day outcome (mRS 3–6) was used as the dependent variable and was also referred to as poor 90-day outcome.

Secondary analyses included ordered logistic regression using the full 90-day mRS score as an ordinal outcome variable, to further evaluate the association between serum Klotho levels and changes across the full functional outcome spectrum.

### 2.5 Statistical analysis

Statistical analysis was performed using R software (version 4.3.2). Continuous variables were expressed as x̄ ± s or M (Q1, Q3) depending on their distribution, with intergroup comparisons performed using independent sample *t*-tests or Mann–Whitney U tests. Categorical variables were expressed as n (%), and comparisons between groups were made using the *χ*^2^ test or Fisher’s exact test. Unfavorable 90-day outcome, defined as 90-day mRS 3–6, was set as the binary dependent variable for logistic regression analyses. Model 1 adjusted for age and sex; Model 2 further adjusted for baseline NIHSS score, atrial fibrillation, diabetes, eGFR, hsCRP, onset-to-blood collection time, and reperfusion therapy. Onset-to-blood collection time was included to account for potential variability in biomarker levels related to differences in the interval between stroke onset and actual serum sampling. Model 3 analyzed serum Klotho according to quartiles and performed trend tests. Restricted cubic spline analysis was used to explore the shape of the association between serum Klotho levels and poor 90-day outcome, including tests for overall and nonlinear associations. ROC curves were used to assess the discriminative ability of the Klotho-alone model, the clinical model, and the combined model, with DeLong test used to compare the differences in AUC. Ordered logistic regression was also performed using the 90-day mRS score as the dependent variable. To assess the potential influence of renal function, fasting status, and sampling-time variability, sensitivity analyses were conducted by excluding patients with CKD history and by restricting the analysis to fasting morning blood samples. Because serum Klotho is closely related to renal function, eGFR was included as an adjustment variable in the main multivariable models, and an additional sensitivity analysis was performed after excluding patients with a history of CKD. Missing data were handled using a complete-case approach. Patients with missing serum Klotho measurement, missing 90-day mRS outcome, or missing key covariates included in the main multivariable models were excluded from the corresponding analyses. Because the final analytic cohort had complete data for the exposure, primary outcome, and main model covariates, multiple imputation was not performed. Results are presented as OR values with *95% CI*, all two-tailed tests, with *p* < 0.05 considered statistically significant. An expanded fully adjusted model was additionally constructed by including ASPECTS score, infarct volume, and pre-stroke mRS score to further assess potential residual confounding.

### 2.6 Quality control

Data quality was ensured by extracting clinical data from the medical records database and follow-up documents by trained research personnel, with double checking by two research personnel. The laboratory test was conducted according to the standard protocol. Data validation was conducted prior to statistical analysis to guarantee accurate analysis results.

## 3 Results

### 3.1 Baseline characteristics

After excluding patients with missing critical clinical data, missing serum Klotho measurement, or unavailable 90-day mRS outcome, 200 patients were included in the final complete-case analysis. Among them, 140 patients had favorable 90-day outcome (mRS 0–2), and 60 patients had unfavorable 90-day outcome (mRS 3–6). Compared with patients with favorable 90-day outcome, those with unfavorable 90-day outcome were older and had more severe neurological deficits, as reflected by higher baseline NIHSS scores and lower ASPECTS scores. They also had lower serum Klotho levels, higher hsCRP levels, larger infarct volumes, longer hospital stays, and higher pre-stroke mRS scores. The proportion of patients receiving endovascular therapy was lower in the unfavorable 90-day outcome group. Other demographic characteristics, vascular risk factors, onset-to-admission time, onset-to-blood collection time, TOAST classification, intravenous thrombolysis, fasting morning sampling status, and chronic kidney disease history were generally comparable between the two groups, as shown in Table 1.

**Table 1:** Baseline characteristics according to 90-day functional outcome.

Characteristic	Overall	Favorable 90-day outcome	Unfavorable 90-day outcome	Statistic	*p*-value
Age, years	67.00 (59.00, 72.00)	66.00 (58.00, 71.00)	70.00 (62.75, 75.25)	*Z* = −2.466	0.014
BMI, kg/m^2^	24.90 (22.60, 26.60)	25.00 (22.87, 26.92)	24.40 (22.50, 26.42)	*Z* = 1.399	0.162
Admission systolic BP, mmHg	152.00 (138.75, 165.00)	151.00 (138.00, 164.00)	157.00 (141.75, 167.50)	*Z* = −1.379	0.168
Admission diastolic BP, mmHg	84.00 (79.00, 92.00)	85.00 (79.00, 95.00)	83.00 (76.00, 86.25)	*Z* = 2.621	0.009
Baseline NIHSS score	10.00 (6.00, 15.00)	8.00 (5.00, 11.00)	16.50 (10.75, 21.25)	*Z* = −6.749	< 0.001
ASPECTS score	9.00 (7.00, 10.00)	9.00 (8.00, 10.00)	7.00 (6.00, 9.00)	*Z* = 5.092	< 0.001
Onset-to-admission time, h	6.70 (4.50, 9.40)	6.75 (4.35, 9.40)	6.60 (4.75, 9.35)	*Z* = −0.336	0.738
Serum Klotho, pg/mL	707.25 (597.50, 789.60)	754.05 (656.00, 817.95)	579.65 (444.50, 664.12)	*Z* = 7.526	< 0.001
Fasting blood glucose, mmol/L	5.60 (4.90, 6.30)	5.60 (4.88, 6.23)	5.50 (5.00, 6.32)	*Z* = −0.839	0.402
Serum creatinine, μmol/L	75.85 (65.95, 86.17)	73.60 (63.75, 82.85)	80.40 (66.73, 88.32)	*Z* = −1.993	0.046
eGFR, mL/min/1.73 m^2^	87.50 (80.02, 95.05)	88.15 (80.27, 95.30)	85.85 (79.25, 94.82)	*Z* = 0.970	0.332
hsCRP, mg/L	3.40 (2.40, 4.73)	3.10 (2.30, 4.40)	3.85 (2.75, 5.88)	*Z* = −2.528	0.012
LDL-C, mmol/L	2.60 (2.21, 3.04)	2.64 (2.27, 3.04)	2.55 (1.90, 3.01)	*Z* = 1.216	0.225
Infarct volume, mL	19.30 (5.20, 37.15)	14.55 (1.38, 28.18)	37.30 (20.03, 65.45)	*Z* = −5.701	< 0.001
Length of hospital stay, days	12.85 (9.60, 15.43)	11.55 (9.10, 14.20)	15.15 (12.07, 19.52)	*Z* = −4.960	< 0.001
Pre-stroke mRS score	0.00 (0.00, 1.00)	0.00 (0.00, 1.00)	1.00 (0.00, 1.00)	*Z* = −2.380	0.017
Onset-to-blood collection time, h	8.60 (6.57, 11.43)	8.60 (6.50, 11.60)	8.55 (6.60, 11.03)	*Z* = −0.217	0.829
Sex				*χ*^2^ = 1.330	0.249
Female	65 (32.5%)	49 (35.0%)	16 (26.7%)		
Male sex	135 (67.5%)	91 (65.0%)	44 (73.3%)		
Hypertension				*χ*^2^ = 0.079	0.778
No	117 (58.5%)	81 (57.9%)	36 (60.0%)		
Yes	83 (41.5%)	59 (42.1%)	24 (40.0%)		
Diabetes mellitus				*χ*^2^ = 0.013	0.909
No	159 (79.5%)	111 (79.3%)	48 (80.0%)		
Yes	41 (20.5%)	29 (20.7%)	12 (20.0%)		
Atrial fibrillation				*χ*^2^ = 0.000	1.000
No	170 (85.0%)	119 (85.0%)	51 (85.0%)		
Yes	30 (15.0%)	21 (15.0%)	9 (15.0%)		
Smoking history				*χ*^2^ = 0.174	0.677
No	146 (73.0%)	101 (72.1%)	45 (75.0%)		
Yes	54 (27.0%)	39 (27.9%)	15 (25.0%)		
Prior stroke history				*χ*^2^ = 0.094	0.759
No	171 (85.5%)	119 (85.0%)	52 (86.7%)		
Yes	29 (14.5%)	21 (15.0%)	8 (13.3%)		
TOAST classification				Fisher	0.889
CE	52 (30.6%)	35 (30.2%)	17 (31.5%)		
LAA	72 (42.4%)	51 (44.0%)	21 (38.9%)		
Other/Undetermined	8 (4.7%)	5 (4.3%)	3 (5.6%)		
SVO	38 (22.4%)	25 (21.6%)	13 (24.1%)		
Intravenous thrombolysis				*χ*^2^ = 0.523	0.470
No	164 (82.0%)	113 (80.7%)	51 (85.0%)		
Yes	36 (18.0%)	27 (19.3%)	9 (15.0%)		
Endovascular therapy				*χ*^2^ = 4.928	0.026
No	161 (80.5%)	107 (76.4%)	54 (90.0%)		
Yes	39 (19.5%)	33 (23.6%)	6 (10.0%)		
Reperfusion treatment				*χ*^2^ = 5.034	0.081
None	132 (66.0%)	87 (62.1%)	45 (75.0%)		
IVT	29 (14.5%)	20 (14.3%)	9 (15.0%)		
EVT ± IVT	39 (19.5%)	33 (23.6%)	6 (10.0%)		
Early neurological deterioration				*χ*^2^ = 0.269	0.604
No	145 (72.5%)	103 (73.6%)	42 (70.0%)		
Yes	55 (27.5%)	37 (26.4%)	18 (30.0%)		
Symptomatic intracerebral hemorrhage				*χ*^2^ = 0.041	0.839
No	165 (82.5%)	115 (82.1%)	50 (83.3%)		
Yes	35 (17.5%)	25 (17.9%)	10 (16.7%)		
Fasting morning blood sample				*χ*^2^ = 1.247	0.264
No	59 (29.5%)	38 (27.1%)	21 (35.0%)		
Yes	141 (70.5%)	102 (72.9%)	39 (65.0%)		
Chronic kidney disease				*χ*^2^ = 1.675	0.196
No	182 (91.0%)	125 (89.3%)	57 (95.0%)		
Yes	18 (9.0%)	15 (10.7%)	3 (5.0%)		

Data are presented as median (interquartile range) or *n* (%), as appropriate. BP, blood pressure; NIHSS, National Institutes of Health Stroke Scale; ASPECTS, Alberta Stroke Program Early CT Score; eGFR, estimated glomerular filtration rate; hsCRP, high-sensitivity C-reactive protein; LDL-C, low-density lipoprotein cholesterol; mRS, modified Rankin Scale; TOAST, Trial of Org 10,172 in Acute Stroke Treatment; IVT, intravenous thrombolysis; EVT, endovascular therapy; END, early neurological deterioration; sICH, symptomatic intracerebral hemorrhage; CKD, chronic kidney disease.

### 3.2 Univariate logistic regression results

Univariate logistic regression analysis revealed that higher serum Klotho levels were significantly associated with a decreased risk of poor 90-day outcome (each 1 standard deviation increase, *OR* = 0.201, *95% CI*: 0.118–0.318, *p* < 0.001). In addition, the following factors were significantly associated with an increased risk of poor 90-day outcome: age (*OR* = 1.046, *95% CI*: 1.013–1.082, *p* = 0.007), baseline NIHSS score (*OR* = 1.201, *95% CI*: 1.136–1.279, *p* < 0.001), hsCRP (*OR* = 1.168, *95% CI*: 1.023–1.342, *p* = 0.024), infarct volume (*OR* = 1.038, *95% CI*: 1.025–1.054, *p* < 0.001), and pre-stroke mRS score (*OR* = 1.651, *95% CI*: 1.078–2.537, *p* = 0.021). On the other hand, lower admission diastolic blood pressure (*OR* = 0.962, *95% CI*: 0.933–0.989, *p* = 0.008), higher ASPECTS scores (*OR* = 0.584, *95% CI*: 0.475–0.704, *p* < 0.001), and endovascular treatment (*OR* = 0.360, *95% CI*: 0.130–0.857, *p* = 0.031) were significantly associated with a reduced risk of poor 90-day outcome. Other variables, including sex, BMI, hypertension, diabetes, atrial fibrillation, smoking history, prior stroke history, admission systolic blood pressure, time from symptom onset to admission, TOAST classification, intravenous thrombolysis, fasting blood glucose, creatinine, eGFR, LDL-C, early neurological deterioration, symptomatic intracerebral hemorrhage, time from symptom onset to blood collection, and chronic kidney disease history, showed no significant association with poor 90-day outcome (all *p* > 0.05), as shown in Table 2.

**Table 2:** Univariate logistic regression analysis of factors associated with poor 90-day outcome in patients with AIS.

Variable	Comparison	*OR* (*95% CI*)	*p-*value
Serum Klotho (per 1 SD increase)	Continuous variable	0.201 (0.118, 0.318)	< 0.001
Age, years	Continuous variable	1.046 (1.013, 1.082)	0.007
Sex	Male vs. female	1.481 (0.769, 2.952)	0.250
BMI, kg/m^2^	Continuous variable	0.914 (0.819, 1.016)	0.101
Hypertension	Yes vs. no	0.915 (0.491, 1.688)	0.778
Diabetes mellitus	Yes vs. no	0.957 (0.438, 1.995)	0.909
Atrial fibrillation	Yes vs. no	1.000 (0.411, 2.274)	1.000
Smoking history	Yes vs. no	0.863 (0.423, 1.700)	0.677
Prior stroke history	Yes vs. no	0.872 (0.344, 2.029)	0.759
Admission systolic BP, mmHg	Continuous variable	1.011 (0.995, 1.027)	0.186
Admission diastolic BP, mmHg	Continuous variable	0.962 (0.933, 0.989)	0.008
Baseline NIHSS score	Continuous variable	1.201 (1.136, 1.279)	< 0.001
ASPECTS score	Continuous variable	0.584 (0.475, 0.704)	< 0.001
Onset-to-admission time, h	Continuous variable	1.013 (0.950, 1.077)	0.689
TOAST classification, LAA vs. CE	LAA vs. CE	0.848 (0.392, 1.845)	0.674
TOAST classification, other/undetermined vs. CE	Other/undetermined vs. CE	1.235 (0.232, 5.654)	0.789
TOAST classification, SVO vs. CE	SVO vs. CE	1.071 (0.437, 2.595)	0.880
Intravenous thrombolysis	Yes vs. no	0.739 (0.309, 1.632)	0.471
Endovascular therapy	Yes vs. no	0.360 (0.130, 0.857)	0.031
Fasting blood glucose, mmol/L	Continuous variable	1.262 (0.909, 1.763)	0.167
Serum creatinine, μmol/L	Continuous variable	1.016 (0.997, 1.035)	0.095
eGFR, mL/min/1.73 m^2^	Continuous variable	0.989 (0.966, 1.013)	0.363
hsCRP, mg/L	Continuous variable	1.168 (1.023, 1.342)	0.024
LDL-C (mmol/L)	Continuous variable	0.708 (0.448, 1.118)	0.141
Infarct volume, mL	Continuous variable	1.038 (1.025, 1.054)	< 0.001
Early neurological deterioration	Yes vs. no	1.180 (0.604, 2.309)	0.629
Symptomatic intracerebral hemorrhage	Yes vs. no	0.926 (0.396, 2.009)	0.849
Pre-stroke mRS score	Continuous variable	1.651 (1.078, 2.537)	0.021
Onset-to-blood collection time, h	Continuous variable	1.010 (0.946, 1.080)	0.755
Chronic kidney disease	Yes vs. no	0.438 (0.099, 1.394)	0.195

### 3.3 Multivariable logistic regression results

Multivariable logistic regression analysis showed that in Model 1, for each 1 standard deviation increase in serum Klotho, the risk of poor 90-day outcome significantly decreased (*OR* = 0.206, *95% CI*: 0.120–0.327, *p* < 0.001). Age and sex were not significantly associated with poor 90-day outcome.

In Model 2, after further adjusting for age, sex, baseline NIHSS score, atrial fibrillation, diabetes, eGFR, hsCRP, time from symptom onset to blood collection, and reperfusion therapy, serum Klotho levels remained independently associated with poor 90-day outcome (each 1 standard deviation increase, *OR* = 0.294, *95% CI*: 0.158–0.505, *p* < 0.001). Additionally, age (*OR* = 1.072, *95% CI*: 1.006–1.148, *p* = 0.037) and baseline NIHSS score (*OR* = 1.161, *95% CI*: 1.071–1.265, *p* < 0.001) were independent risk factors for poor 90-day outcome. Endovascular therapy ± intravenous thrombolysis was significantly associated with a reduced risk of poor 90-day outcome (*OR* = 0.199, *95% CI*: 0.049–0.651, *p* = 0.013).

In Model 3, using the lowest serum Klotho quartile (Q1) as the reference, the Q3 group (*OR* = 0.171, *95% CI*: 0.047–0.558, *p* = 0.005) and Q4 group (*OR* = 0.073, *95% CI*: 0.010–0.352, *p* = 0.003) were significantly associated with a decreased risk of poor 90-day outcome, whereas the Q2 group showed no significant difference (*OR* = 0.512, *95% CI*: 0.191–1.350, *p* = 0.178). Trend testing showed a significant decrease in the risk of poor 90-day outcome with increasing Klotho quartiles (*OR* = 0.414, *95% CI*: 0.253–0.656, *p* < 0.001). Moreover, in both Model 3 and trend testing, age, baseline NIHSS score, and endovascular therapy ± intravenous thrombolysis remained statistically significant, as shown in Table 3.

**Table 3:** Multivariable logistic regression analysis of factors associated with poor 90-day outcome.

Variable	*OR* (*95% CI*)	*p-*value
Model 1 (minimal adjustment: Klotho + Age + Sex)		
Serum Klotho (per 1 SD increase)	0.206 (0.120, 0.327)	< 0.001
Age, years	1.036 (0.995, 1.082)	0.094
Male sex	1.377 (0.621, 3.162)	0.438
Model 2 (clinical adjustment model)		
Serum Klotho (per 1 SD increase)	0.294 (0.158, 0.505)	< 0.001
Age, years	1.072 (1.006, 1.148)	0.037
Male sex	1.324 (0.544, 3.331)	0.541
Baseline NIHSS score	1.161 (1.071, 1.265)	< 0.001
Atrial fibrillation	1.276 (0.371, 4.195)	0.691
Diabetes mellitus	0.979 (0.341, 2.693)	0.968
eGFR, mL/min/1.73 m^2^	1.018 (0.971, 1.069)	0.459
hsCRP, mg/L	0.941 (0.742, 1.159)	0.593
Onset-to-blood collection time, h	0.993 (0.902, 1.091)	0.891
Intravenous thrombolysis (vs none)	0.882 (0.218, 3.361)	0.855
Endovascular therapy ± intravenous thrombolysis (vs none)	0.199 (0.049, 0.651)	0.013
Model 3 (Klotho quartiles)		
Serum Klotho Q2 vs. Q1	0.512 (0.191, 1.350)	0.178
Serum Klotho Q3 vs. Q1	0.171 (0.047, 0.558)	0.005
Serum Klotho Q4 vs. Q1	0.073 (0.010, 0.352)	0.003
Age, years	1.072 (1.006, 1.148)	0.033
Male sex	1.393 (0.566, 3.430)	0.488
Baseline NIHSS score	1.166 (1.075, 1.267)	< 0.001
Atrial fibrillation	1.615 (0.504, 5.160)	0.41
Diabetes mellitus	0.910 (0.322, 2.480)	0.856
eGFR, mL/min/1.73 m^2^	1.014 (0.967, 1.060)	0.559
hsCRP, mg/L	0.942 (0.747, 1.160)	0.595
Onset-to-blood collection time, h	0.979 (0.889, 1.070)	0.65
Intravenous thrombolysis (vs none)	0.861 (0.216, 3.290)	0.828
Endovascular therapy ± intravenous thrombolysis (vs none)	0.221 (0.061, 0.679)	0.013
Trend test analysis of factors associated with poor 90-day outcome		
Serum Klotho quartile trend	0.414 (0.253, 0.656)	< 0.001
Age, years	1.072 (1.006, 1.148)	0.035
Male sex	1.390 (0.574, 3.460)	0.473
Baseline NIHSS score	1.166 (1.076, 1.265)	< 0.001
Atrial fibrillation	1.576 (0.493, 4.980)	0.432
Diabetes mellitus	0.900 (0.319, 2.440)	0.838
eGFR, mL/min/1.73 m^2^	1.014 (0.967, 1.060)	0.568
hsCRP, mg/L	0.943 (0.747, 1.160)	0.6
Onset-to-blood collection time, h	0.982 (0.894, 1.080)	0.702
Intravenous thrombolysis (vs none)	0.867 (0.218, 3.260)	0.834
Endovascular therapy ± intravenous thrombolysis (vs none)	0.219 (0.060, 0.676)	0.013

OR represents the odds ratio, and CI represents the 95% confidence interval. Model 1 is the minimal adjustment model; Model 2 includes Klotho, age, sex, baseline NIHSS score, atrial fibrillation, diabetes, eGFR, hsCRP, onset-to-blood collection time, and reperfusion therapy; Model 3 uses the lowest Klotho quartile (Q1) as the reference, and the trend test incorporates Klotho quartiles as an ordinal variable in the model.

To further address potential residual confounding, an expanded fully adjusted model was constructed by additionally including ASPECTS score, infarct volume, and pre-stroke mRS score. In this expanded model, serum Klotho remained independently associated with poor 90-day outcome. Each 1-SD increase in serum Klotho was associated with a lower risk of poor 90-day outcome (*OR* = 0.247, *95% CI*: 0.131–0.467, *p* < 0.001). ASPECTS score was also independently associated with poor 90-day outcome (*OR* = 0.585, *95% CI*: 0.387–0.885, *p* = 0.011). In contrast, infarct volume and pre-stroke mRS score were not statistically significant after adjustment for other covariates.

Multicollinearity diagnostics showed that all VIF values were below 5, suggesting no serious multicollinearity. The Hosmer–Lemeshow test indicated acceptable model fit (*χ*^2^ = 3.793, df = 8, *p* = 0.875). The AIC, BIC, and Nagelkerke pseudo-*R*^2^ of the expanded model were 169.073, 218.548, and 0.580, respectively.

### 3.4 Restricted cubic splines

**Figure 1:**
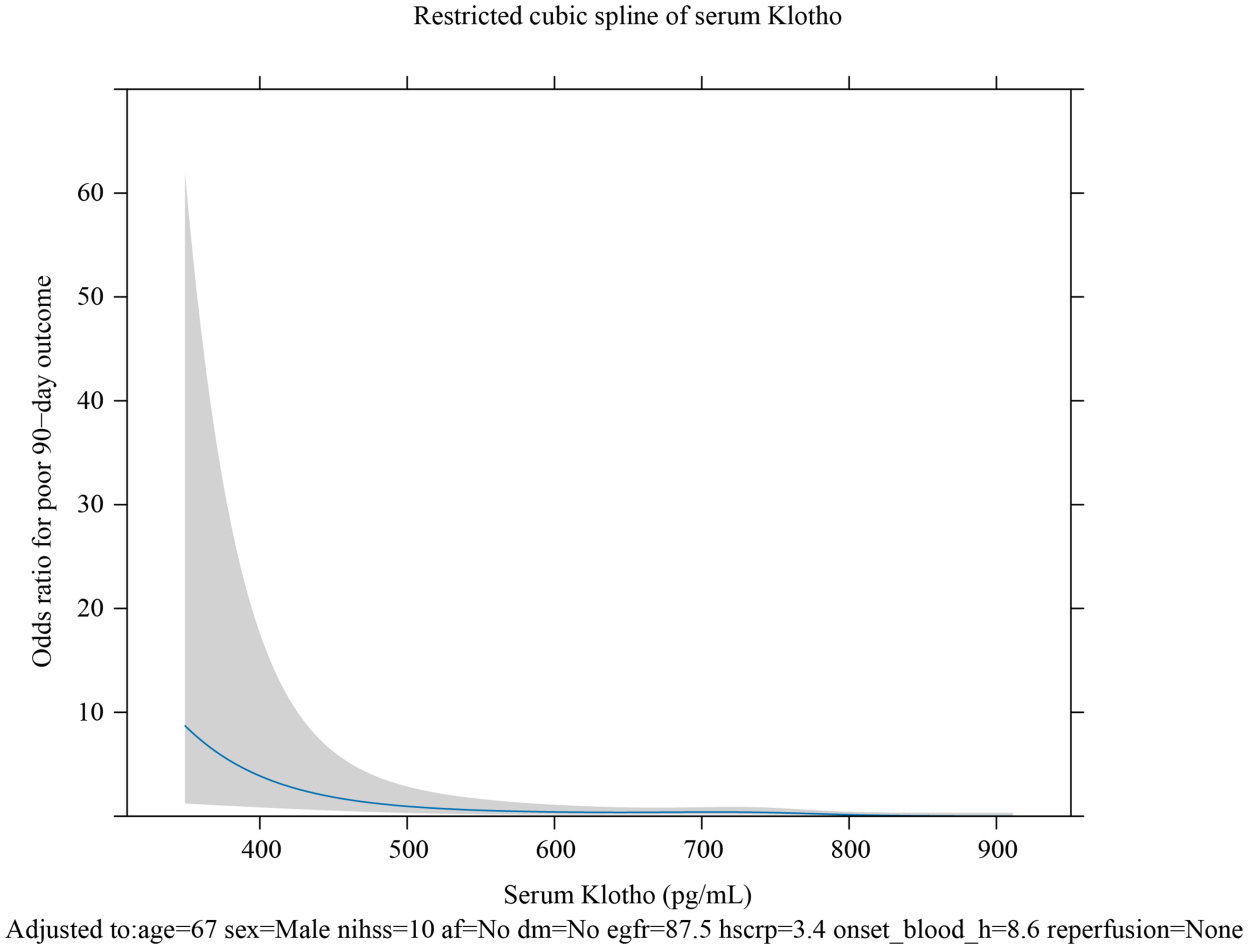
Restricted cubic spline analysis of serum Klotho levels and poor 90-day outcome in patients with AIS. The model was adjusted for age, sex, baseline NIHSS score, atrial fibrillation, diabetes, eGFR, hsCRP, onset-to-blood collection time, and reperfusion therapy. The analysis showed an overall inverse association between serum Klotho and poor 90-day outcome, without evidence of significant nonlinearity.

Restricted cubic spline analysis was performed to explore the shape of the association between serum Klotho levels and poor 90-day outcome. After adjustment for age, sex, baseline NIHSS score, atrial fibrillation, diabetes, eGFR, hsCRP, onset-to-blood collection time, and reperfusion therapy, serum Klotho showed a significant overall association with poor 90-day outcome. However, the test for nonlinearity was not statistically significant, indicating no clear evidence of a nonlinear relationship. Therefore, the RCS analysis mainly supported an overall inverse association between serum Klotho levels and poor 90-day outcome rather than a distinct nonlinear dose–response pattern, as shown in Table 4 and Figure 1.

**Table 4:** Restricted cubic spline analysis of serum Klotho and poor 90-day outcome.

Variable	*χ*^2^ value	df	*p-*value
Serum Klotho, overall association	15.42	3	0.0015
Nonlinear	3.33	2	0.1887
Age, years	4.55	1	0.0329
Sex	0.32	1	0.574
Baseline NIHSS score	13.88	1	0.0002
Atrial fibrillation	0.03	1	0.8545
Diabetes mellitus	0.02	1	0.8765
eGFR, mL/min/1.73 m^2^	0.51	1	0.474
hsCRP, mg/L	0.56	1	0.4532
Onset-to-blood collection time, h	0.02	1	0.8992
Reperfusion treatment	5.82	2	0.0545
Overall model	40.08	13	0.0001

The restricted cubic spline model adjusted for variables including age, sex, baseline NIHSS score, atrial fibrillation, diabetes, eGFR, hsCRP, time from symptom onset to blood collection, and reperfusion therapy. “Nonlinear” represents the test for the nonlinear component of serum Klotho.

### 3.5 ROC curve analysis

**Figure 2:**
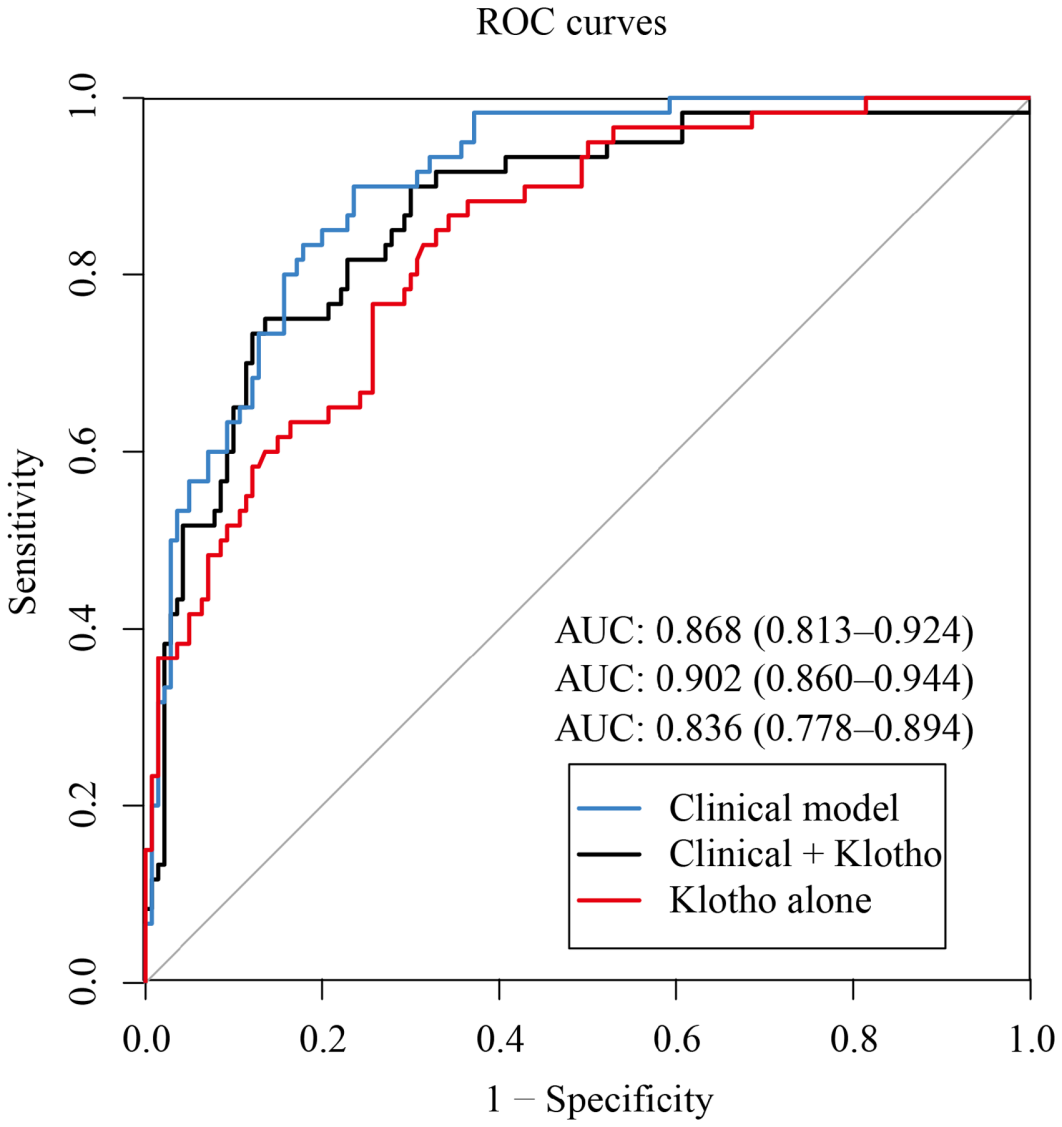
ROC curves for predicting poor 90-day outcome in AIS patients using different models. The clinical + Klotho model showed a numerically higher AUC than the clinical model alone; however, this improvement was not statistically significant according to the DeLong test.

In ROC analysis, the AUCs of the Klotho-alone model, clinical model, and clinical + Klotho model were 0.836, 0.868, and 0.902, respectively. Although the combined model showed a numerically higher AUC than the clinical model alone, the DeLong test did not show a statistically significant difference between the two models. These findings suggest that serum Klotho may have prognostic relevance, but its incremental discriminative value beyond established clinical variables was limited in the present sample, as shown in Table 5 and Figure 2.

**Table 5:** ROC curve analysis results.

Model	AUC	*95% CI*
Klotho-alone model	0.836	0.778–0.894
Clinical model	0.868	0.813–0.924
Clinical + Klotho model	0.902	0.860–0.944

AUC represents the area under the receiver operating characteristic (ROC) curve; 95% CI represents the 95% confidence interval. DeLong test comparing the clinical model and the combined clinical + Klotho model: *Z* = −1.601, *p* = 0.109.

### 3.6 Ordered logistic regression analysis and results description

Ordered logistic regression was further performed using the full 90-day mRS score as an ordinal outcome. After adjustment for serum Klotho, age, sex, baseline NIHSS score, atrial fibrillation, diabetes, eGFR, hsCRP, onset-to-blood collection time, and reperfusion therapy, age and baseline NIHSS score remained significantly associated with worse functional outcome. Specifically, each 1-year increase in age was associated with higher odds of a worse mRS category, and each 1-point increase in baseline NIHSS score was also associated with higher odds of worse functional disability.

In contrast, serum Klotho was not significantly associated with the full ordinal mRS distribution. This finding differed from the binary logistic regression analysis based on mRS 0–2 versus 3–6. Therefore, the ordered logistic regression did not support a consistent association between serum Klotho and incremental shifts across all mRS categories, as shown in Table 6.

**Table 6:** Ordered logistic regression analysis of factors associated with full-scale 90-day mRS scores.

Variable	*OR* (*95% CI*)	Statistic	*p*-value
Serum Klotho (per 1 SD increase)	1.176 (0.755–1.836)	*Z* = 0.715	0.474
Age, years	1.058 (1.013–1.106)	*Z* = 2.530	0.011
Sex, male vs. female	0.912 (0.513–1.622)	*Z* = −0.312	0.755
Baseline NIHSS score	1.244 (1.158–1.340)	*Z* = 5.873	< 0.001
Atrial fibrillation, yes vs. no	1.267 (0.580–2.767)	*Z* = 0.595	0.552
Diabetes mellitus, yes vs. no	1.164 (0.598–2.259)	*Z* = 0.449	0.654
eGFR(mL/min/1.73 m^2^)	1.001 (0.970–1.034)	*Z* = 0.081	0.936
hsCRP(mg/L)	0.973 (0.856–1.107)	*Z* = −0.420	0.674
Onset-to-blood collection time, h	0.959 (0.895–1.027)	*Z* = −1.199	0.23
Reperfusion treatment, IVT vs. none	0.521 (0.231–1.164)	*Z* = −1.586	0.113
Reperfusion treatment, EVT ± IVT vs. none	0.517 (0.261–1.016)	*Z* = −1.908	0.056

OR represents the odds ratio; 95% CI represents the 95% confidence interval; reperfusion treatment is referenced as “no reperfusion treatment.” The threshold parameters for the proportional odds model are not shown in this table.

### 3.7 Sensitivity analysis

In the sensitivity analysis excluding patients with a history of CKD, the association between serum Klotho and poor 90-day outcome remained statistically significant. Specifically, each 1-SD increase in serum Klotho was associated with a lower risk of poor 90-day outcome (*OR* = 0.301, *95% CI*: 0.161–0.520, *p* < 0.001). Baseline NIHSS score remained independently associated with poor 90-day outcome (*OR* = 1.148, *95% CI*: 1.058–1.252, *p* = 0.001). Endovascular therapy with or without intravenous thrombolysis was also associated with a reduced risk of poor 90-day outcome (*OR* = 0.176, *95% CI*: 0.041–0.598, *p* = 0.010).

To address potential variability related to fasting status and sampling time, an additional sensitivity analysis was restricted to patients with fasting morning blood samples. The association between serum Klotho and poor 90-day outcome remained statistically significant. For each 1-SD increase in serum Klotho, the risk of poor 90-day outcome decreased by 67.6% (*OR* = 0.324, *95% CI*: 0.151–0.632, *p* = 0.002). Age and baseline NIHSS score remained independent risk factors, and endovascular therapy ± intravenous thrombolysis continued to show a protective association.

Overall, both sensitivity analyses showed effect directions generally consistent with the main analysis, suggesting that the association between serum Klotho levels and poor 90-day outcome was robust across these additional analyses, as shown in Tables 7, 8.

**Table 7:** Association between serum Klotho and poor 90-day outcome in sensitivity analyses.

Sensitivity analysis	Variable	*OR* (*95% CI*)	*p*-value
Excluding patients with a history of CKD	Serum Klotho (per 1 SD increase)	0.301 (0.161, 0.520)	< 0.001
	Age, years	1.055 (0.988, 1.131)	0.119
	Baseline NIHSS score	1.148 (1.058, 1.252)	0.001
Restricting to fasting morning blood samples	Serum Klotho (per 1 SD increase)	0.324 (0.151, 0.632)	0.002
	Age, years	1.131 (1.043, 1.241)	0.005
	Baseline NIHSS score	1.204 (1.093, 1.345)	< 0.001

**Table 8:** Multivariable logistic regression results from sensitivity analyses.

Variable	Excluding patients with CKD, *OR* (*95% CI*)	*p-*value	Fasting morning samples only, *OR* (*95% CI*)	*p-*value
Serum Klotho (per 1 SD increase)	0.301 (0.161, 0.520)	<0.001	0.324 (0.151, 0.632)	0.002
Age, years	1.055 (0.988, 1.131)	0.119	1.131 (1.043, 1.241)	0.005
Male sex	1.158 (0.466, 2.967)	0.753	1.394 (0.448, 4.674)	0.574
Baseline NIHSS score	1.148 (1.058, 1.252)	0.001	1.204 (1.093, 1.345)	< 0.001
Atrial fibrillation	1.344 (0.363, 4.746)	0.648	2.020 (0.428, 9.371)	0.362
Diabetes mellitus	0.960 (0.328, 2.690)	0.940	2.210 (0.670, 7.475)	0.192
eGFR, mL/min/1.73 m^2^	1.003 (0.952, 1.057)	0.909	1.050 (0.991, 1.117)	0.111
hsCRP, mg/L	0.967 (0.760, 1.195)	0.769	0.874 (0.639, 1.139)	0.361
Onset-to-blood collection time, h	0.992 (0.892, 1.099)	0.885	0.960 (0.855, 1.071)	0.474
Intravenous thrombolysis (vs none)	0.900 (0.219, 3.531)	0.88	0.909 (0.150, 5.084)	0.915
Endovascular therapy ± intravenous thrombolysis (vs none)	0.176 (0.041, 0.598)	0.01	0.067 (0.002, 0.517)	0.037

Given the potential influence of renal function on circulating Klotho levels, we performed a sensitivity analysis after excluding patients with a history of CKD. The association between serum Klotho and poor 90-day outcome remained statistically significant, suggesting that the main finding was not solely driven by patients with known CKD. However, residual confounding related to renal function could not be completely excluded.

### 3.8 Forest plot

**Figure 3:**
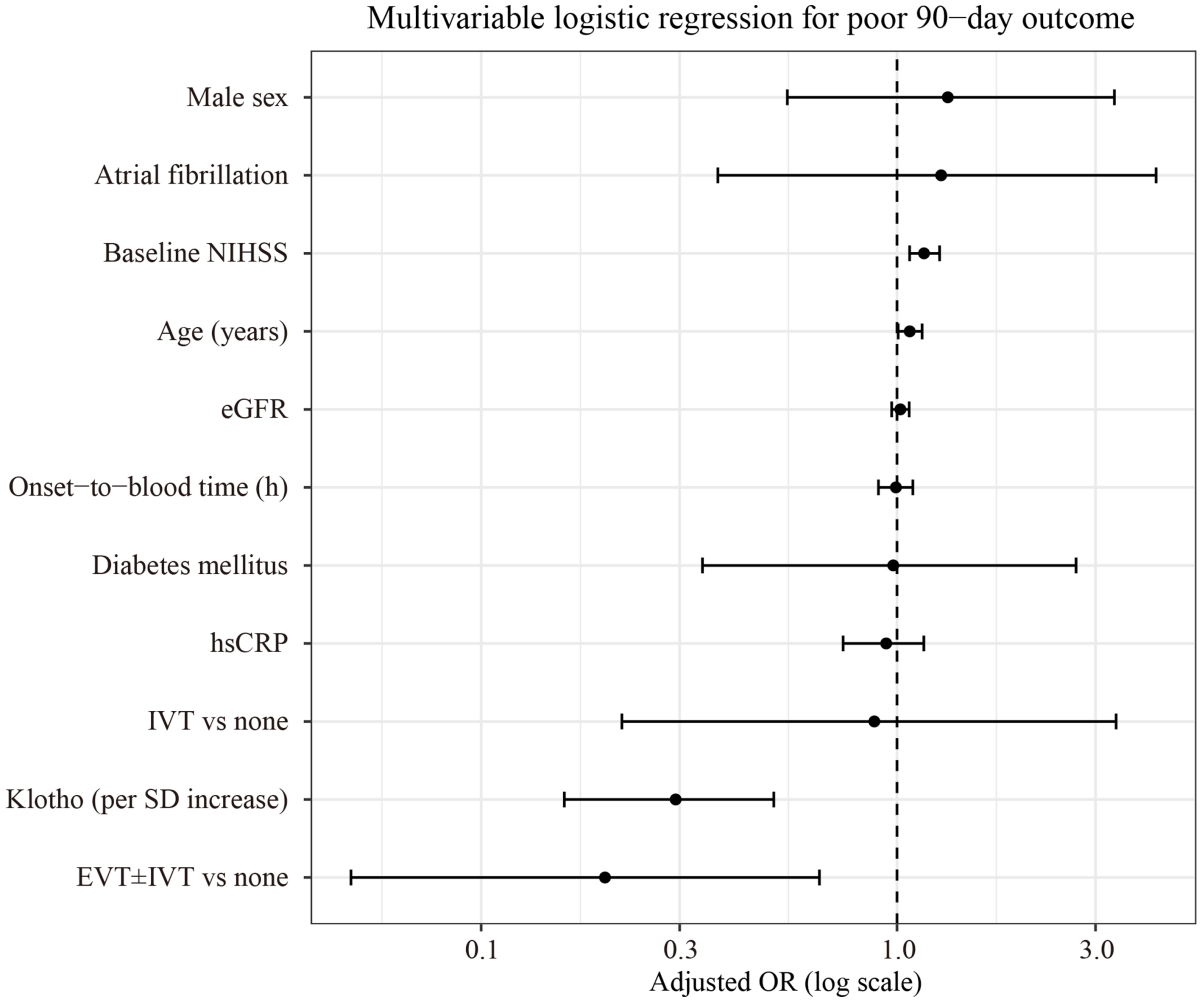
Forest plot for multivariable logistic regression analysis of poor 90-day outcome. The forest plot shows the *OR*s and 95% confidence intervals (*CI*s) of variables included in Model 2 for poor 90-day outcome in patients with AIS. The vertical reference line corresponds to *OR* = 1. Variables associated with poor 90-day outcome included serum Klotho, age, baseline NIHSS score, and reperfusion treatment.

The forest plot further illustrated the effect estimates of variables included in the multivariable logistic regression model for poor 90-day outcome. Higher serum Klotho levels were associated with a lower risk of poor 90-day outcome, whereas older age and higher baseline NIHSS scores were positively associated with poor 90-day outcome. Endovascular therapy with or without intravenous thrombolysis was associated with a reduced risk of poor 90-day outcome. Most other variables had *95% CI*s crossing 1, indicating no statistically significant independent association with poor 90-day outcome, as shown in Figure 3.

## 4 Discussion

The present study showed that serum Klotho levels were significantly lower in patients with unfavorable 90-day outcome than in those with favorable 90-day outcome. After adjustment for age, sex, baseline NIHSS score, atrial fibrillation, diabetes mellitus, eGFR, hsCRP, onset-to-blood collection time, and reperfusion treatment, serum Klotho remained independently associated with poor 90-day outcome, defined as 90-day mRS 3–6. This association was consistent when serum Klotho was analyzed as a continuous variable, by quartiles, and in trend testing. RCS analysis showed a significant overall association, but no clear evidence of a nonlinear association. Sensitivity analyses excluding patients with CKD history and restricting to fasting morning blood samples yielded generally consistent results. However, ROC analysis showed that adding serum Klotho to the clinical model increased the AUC numerically from 0.868 to 0.902, but this improvement was not statistically significant according to the DeLong test.

An important issue in the present study is the discrepancy between the binary and ordinal outcome analyses. Serum Klotho was significantly associated with poor 90-day outcome when mRS was dichotomized as 0–2 versus 3–6, but this association was not significant in the ordered logistic regression using the full mRS scale. This finding indicates that the prognostic relevance of serum Klotho should not be interpreted as extending uniformly across the entire functional disability spectrum.

Several explanations may account for this discrepancy. First, the dichotomized mRS endpoint captures a clinically meaningful transition from functional independence to dependency or death, whereas the ordinal mRS model evaluates shifts across all disability categories. A biomarker may be associated with the occurrence of clinically defined poor outcome without necessarily discriminating among adjacent mRS categories. Second, the ordered logistic model requires stronger assumptions, including a consistent proportional effect across mRS thresholds, which may not hold for serum Klotho. Third, the moderate sample size and relatively small number of patients in some mRS categories may have limited the statistical power of the ordinal analysis. Finally, age and baseline NIHSS score may explain a larger proportion of the variation in the full mRS distribution, thereby attenuating the independent contribution of serum Klotho in the ordinal model.

Therefore, our findings support an association between lower serum Klotho levels and dichotomized poor 90-day outcome, but they do not establish serum Klotho as a robust marker of the full range of post-stroke functional disability. This limitation has been incorporated into the interpretation of the results.

The ROC and RCS findings should also be interpreted cautiously. Although the clinical + Klotho model had a numerically higher AUC than the clinical model alone, the DeLong test did not demonstrate a statistically significant improvement. Therefore, the present study does not provide sufficient evidence that serum Klotho meaningfully improves risk discrimination beyond established clinical predictors such as age, baseline NIHSS score, and reperfusion therapy. Similarly, although the RCS analysis showed an overall association between serum Klotho and poor 90-day outcome, the nonlinear component was not statistically significant. Thus, the spline analysis should be interpreted as supporting a general inverse association rather than a specific nonlinear dose–response relationship. These findings suggest that serum Klotho may be associated with short-term functional outcome after AIS, but its incremental predictive value and biological interpretation require further validation.

Our findings should be interpreted in the context of emerging but still limited evidence on circulating Klotho and cerebrovascular disease. A recent population-based NHANES study reported an inverse association between serum Klotho levels and the prevalence of stroke, but its cross-sectional design precluded causal inference (14). In a retrospective study of young to middle-aged patients with AIS, Klotho levels were lower in AIS patients than in stroke-free controls and were inversely associated with AIS risk, suggesting its potential relevance to early risk stratification rather than established post-stroke prognosis (12). In addition, serum Klotho has been inversely associated with C-reactive protein in a large population-based analysis, supporting a possible link between Klotho and systemic inflammatory status; however, this evidence is not stroke-specific and should therefore be interpreted cautiously (13). Taken together, these studies suggest that lower circulating Klotho may reflect vascular vulnerability, systemic inflammation, or overall biological aging. However, evidence directly evaluating serum Klotho as a prognostic biomarker for functional outcome after AIS remains insufficient. Therefore, the present study adds preliminary clinical evidence that early serum Klotho levels are associated with 90-day functional outcome, while larger prospective studies with repeated measurements are needed to clarify its temporal stability, incremental predictive value, and biological significance.

From a mechanistic perspective, the association between serum Klotho and AIS outcome should be interpreted cautiously. Recent experimental and review evidence suggests that Klotho may participate in neuroinflammatory regulation, oxidative stress modulation, and ischemic brain injury responses, but most of this evidence comes from preclinical models or broad neurodegenerative contexts rather than prospective human AIS cohorts (17, 18). For example, an experimental study suggested that STAT4-mediated Klotho up-regulation may contribute to brain ischemic tolerance by inhibiting neuronal pyroptosis (17). A recent review also summarized the potential anti-inflammatory and antioxidant roles of soluble Klotho in brain aging and ischemic stroke-related conditions (18). However, the present observational study cannot determine whether lower serum Klotho is causally involved in post-stroke injury or whether it reflects older age, renal function, systemic inflammation, vascular vulnerability, or overall disease severity. Accordingly, serum Klotho should be regarded as an early circulating biomarker associated with short-term functional outcome rather than direct evidence of a specific mechanistic pathway after AIS.

Several limitations should be acknowledged. First, this was a single-center observational study with a moderate sample size, and the number of unfavorable outcome events was limited; therefore, the stability of effect estimates may be affected. Second, serum Klotho was measured only once using a blood sample obtained within 24 h after admission. Therefore, intra-individual variability and temporal trajectories of serum Klotho during the acute and recovery phases of AIS could not be evaluated. The present findings should be interpreted as reflecting the association between early circulating Klotho levels and 90-day outcome rather than the biological effect of dynamic Klotho changes after stroke. In addition, acute physiological stress, systemic inflammation, and renal function may influence serum Klotho concentrations. Although eGFR was adjusted for in the multivariable models and sensitivity analysis excluding patients with CKD yielded consistent results, residual confounding related to renal function and acute stress responses cannot be completely excluded. Future prospective studies with repeated Klotho measurements are needed to clarify intra-individual variability, assay stability over time, and temporal patterns of Klotho after AIS. Third, because AIS is an acute medical condition, blood samples could not be collected under identical fasting, circadian, and onset-to-sampling conditions for all patients. Although all Klotho samples were collected within 24 h after hospital admission, the interval from stroke onset to actual blood collection varied among patients. We therefore recorded onset-to-blood collection time and adjusted for it in the multivariable models. In addition, the sensitivity analysis restricted to fasting morning samples yielded generally consistent results. Nevertheless, residual variability related to fasting status, circadian rhythm, and onset-to-sampling time cannot be completely excluded. Fourth, although the clinical + Klotho model showed a numerically higher AUC than the clinical model alone, the DeLong test was not statistically significant. Therefore, the incremental predictive value of serum Klotho requires confirmation in larger multicenter prospective studies. In addition, the exclusion of patients with severe infections, malignancies, autoimmune diseases, or other conditions that may substantially affect serum Klotho levels may have introduced selection bias. Although these criteria were used to reduce potential confounding related to systemic diseases influencing circulating Klotho concentrations, they may also have resulted in a relatively selected study population and limited the generalizability of our findings to broader real-world AIS populations with complex comorbidities. Future multicenter studies with broader inclusion criteria are needed to validate the prognostic relevance of serum Klotho in more representative stroke cohorts.

In this single-center retrospective observational cohort study, lower early serum Klotho levels were independently associated with unfavorable 90-day outcome, defined as mRS 3–6, in patients with AIS. However, the addition of serum Klotho to established clinical variables did not significantly improve model discrimination, and RCS analysis did not show evidence of a significant nonlinear association. These findings suggest that early serum Klotho may be a candidate biomarker associated with short-term functional outcome after AIS, but its incremental predictive value, temporal stability, and biological significance require further confirmation in larger prospective studies with repeated biomarker measurements.

## Data Availability

The datasets that support the findings of this study are available from the corresponding author upon reasonable request. The datasets are not publicly available because they contain clinical information involving human participants.
