# Supplementary material for: Serum Klotho as a potential biomarker for prognosis in acute ischemic stroke
Source: Front Med (Lausanne). 2026 Jun 3;13:1850285. doi: 10.3389/fmed.2026.1850285 (PMC13273123; doi:10.3389/fmed.2026.1850285)
Supplement: Supplementary file 1 [file Data_Sheet_1.pdf]

Supplementary Table S1. Expanded multivariable logistic regression model for poor 90-day outcome and multicollinearity diagnostics

**Variable**	**β**	**SE**	***OR***	***95% CI***	***P*-value**	**VIF**
Serum Klotho, per 1 SD increase	-1.399	0.325	0.247	0.131–0.467	<0.001	1.84
Age, years	0.088	0.036	1.092	1.017–1.171	0.015	2.54
Male sex	0.337	0.472	1.4	0.555–3.531	0.476	1.07
Baseline NIHSS score	0.058	0.063	1.059	0.936–1.199	0.363	4.59
ASPECTS score	-0.536	0.211	0.585	0.387–0.885	0.011	3.41
Infarct volume, mL	-0.005	0.016	0.995	0.965–1.027	0.773	4.38
Pre-stroke mRS score	0.121	0.313	1.129	0.611–2.086	0.698	1.11
Atrial fibrillation	-0.14	0.653	0.869	0.242–3.124	0.83	1.14
Diabetes mellitus	-0.012	0.543	0.988	0.341–2.861	0.982	1.09
eGFR, mL/min/1.73 m^2	0.018	0.025	1.018	0.969–1.070	0.482	2.4
hsCRP, mg/L	-0.066	0.122	0.936	0.737–1.189	0.587	1.33
Onset-to-blood collection time, h	0.012	0.05	1.012	0.917–1.117	0.81	1.24
IVT vs none	-0.301	0.747	0.74	0.171–3.200	0.687	1.25
EVT ± IVT vs none	-1.76	0.669	0.172	0.046–0.638	0.009	1.1

Note：The expanded model additionally included ASPECTS score, infarct volume, and pre-stroke mRS score on the basis of the clinical adjustment model. *OR*, odds ratio; *CI*, confidence interval; VIF, variance inflation factor; NIHSS, National Institutes of Health Stroke Scale; ASPECTS, Alberta Stroke Program Early CT Score; mRS, modified Rankin Scale; eGFR, estimated glomerular filtration rate; hsCRP, high-sensitivity C-reactive protein; IVT, intravenous thrombolysis; EVT, endovascular therapy.

Supplementary Table S2. Model performance and goodness-of-fit statistics for the expanded multivariable logistic regression model

Metric	Expanded model
AIC	169.073
BIC	218.548
McFadden pseudo-R^2	0.431
Nagelkerke pseudo-R^2	0.58
Apparent AUC	0.91
Hosmer–Lemeshow P	0.875
Maximum VIF	4.59

Note：AIC, Akaike information criterion; BIC, Bayesian information criterion; AUC, area under the receiver operating characteristic curve; VIF, variance inflation factor. The Hosmer–Lemeshow test was used to assess model calibration, with $P > 0.05$ indicating no evidence of poor fit.
